# O28 A rare sight-threatening complication of a rare disease

**DOI:** 10.1093/rap/rkab067.027

**Published:** 2021-10-19

**Authors:** Mithun Chakravorty, Archana Pradeep, Ira Pande

**Affiliations:** Nottingham University Hospital, Nottingham, United Kingdom

## Abstract

**Case report - Introduction:**

Relapsing polychondritis (RP) is a rare autoimmune disorder characterised by inflammation of cartilaginous structures throughout the body. It usually presents in the fourth and sixth decade, and commonly affected areas include the nasal and respiratory tracts, external ears and joints. Ocular involvement is reported in around 65% of RP patients during their lifetime but is rarely sight-threatening. However, we present an unusual case of recurrent ocular inflammation due to RP that resulted in unilateral posterior scleritis with sub-retinal exudation, and posed a high risk of retinal detachment. Prompt escalation of immunosuppressive treatment was required to prevent this.

**Case report - Case description:**

A 48-year-old man of south-east Asian descent presented to rheumatology in December 2020 with a typical history of new inflammatory arthritis of 4 weeks duration. He was known to have bilateral episcleritis and ocular hypertension for 3 years and took Brinzolamide and Latanoprost eye drops, as well as metformin for type 2 diabetes mellitus. The only other relevant history was treatment with antibiotics as an inpatient for bilateral pinna cellulitis 2 months prior, which was suspected to be related to his diabetes. Examination revealed mildly reduced hand grips but no definite synovitis. The most remarkable finding was bilateral painless red eyes. Both pinnae appeared inflamed on close inspection without obvious auricular lobe involvement. Blood tests showed raised C-Reactive Protein (38 mg/L), and Erythrocyte Sedimentation Rate (62 mm/hr), with normochromic normocytic anaemia but other blood counts were normal. Renal and liver function was normal. Detailed immunology was negative.

Relapsing Polychondritis was suspected and confirmed following multidisciplinary team (MDT) discussions with the Ear Nose and Throat and ophthalmology teams. At the next visit, his disease progressed to florid polyarthritis in a rheumatoid distribution. This responded well to low-dose prednisolone and methotrexate. However, the patient attended eye casualty on multiple occasions over the next 6 weeks with alternating acute eye pain and redness, mildly reduced visual acuity and raised intraocular pressures. He developed sub-retinal fluid pockets suggestive of posterior scleritis in the right eye and retinal tomography demonstrated extensive pigment clumping especially in the macula with outer retinal disruption. Urgent pulses of intravenous methylprednisolone were given, followed by high-dose prednisolone tapering. The case was discussed with the regional uveitis MDT, and treatment was escalated to adalimumab. He is currently 1 month into treatment and has preserved visual acuity and stable intraocular pressures, with no other systemic involvement of his RP.

**Case report - Discussion:**

The diagnosis of RP requires a high index of clinical suspicion given the lack of diagnostic markers. The clinical features are multisystem and might not occur simultaneously as in this case, where the eye disease preceded the ear and joint manifestations by a couple of years. Perichondritis might be more difficult to appreciate in patients with darker skin and can further delay diagnosis. The average time to diagnosis from the initial symptoms has been reported as 14 months from a case series of 158 patients with RP in China.

Ocular complications are usually bilateral and the commonest are episcleritis and scleritis (over 50% of cases have one of these), followed by uveitis and retinopathy. Eyelid oedema, proptosis and optic neuritis can also occur but are rarer, and exudative retinal detachment has only been noted in a small number of case reports. Unfortunately, no current treatment guidelines exist for RP due to a lack of randomised controlled trials but treatment is usually guided by the severity of manifestations in conjunction with trends from case series and expert opinion. The aim is to halt or slow disease progression and glucocorticoids are often used first-line for moderate to severe disease, with or without the addition of steroid-sparing agents such as methotrexate, ciclosporin and azathioprine. A meta-analysis of biologics in RP conducted by Kemta et al. in 2012 has suggested anti-TNF (particularly Infliximab) might be beneficial as second-line treatment for severe or refractory cases of organ involvement due to RP such as central nervous system, nasal or pulmonary involvement. However, due to the extreme rarity of both RP and associated severe retinal disease, the optimum therapeutic choice in this setting is currently not known.

**Case report - Key learning points:**

- RP remains a challenging clinical diagnosis with a common delay in diagnosis of more than 1 year from the onset of symptoms. High index of suspicion and MDT input can aid earlier diagnosis and treatment to mitigate further complications.
- Eye disease occurs in up to two-thirds of patients with RP but exudative retinal or subretinal involvement is very rare. It can occur even when other manifestations of RP are well-controlled with immunosuppression as in this case.
- Anti-TNF treatment could be used to minimise visual loss in these severe cases of inflammatory eye disease due to RP, given reported benefit for other organ-threatening complications of RP. However, future case series are needed to guide evidence-based treatment choices.

